# Biochemical characterization of specific Alanine Decarboxylase (AlaDC) and its ancestral enzyme Serine Decarboxylase (SDC) in tea plants (*Camellia sinensis*)

**DOI:** 10.1186/s12896-021-00674-x

**Published:** 2021-03-01

**Authors:** Peixian Bai, Liyuan Wang, Kang Wei, Li Ruan, Liyun Wu, Mengdi He, Dejiang Ni, Hao Cheng

**Affiliations:** 1 National Center for Tea Improvement, Tea Research Institute Chinese Academy of Agricultural Sciences (TRICAAS), 9 Meiling South Road, Hangzhou, 310008 Zhejiang China; 2 College of Horticulture and Forestry Sciences, Huazhong Agricultural University, Wuhan, 430070 Hubei China

**Keywords:** Alanine decarboxylase (AlaDC), Serine decarboxylase (SDC), *Camellia sinensis*, Biochemical properties

## Abstract

**Background:**

Alanine decarboxylase (AlaDC), specifically present in tea plants, is crucial for theanine biosynthesis. Serine decarboxylase (SDC), found in many plants, is a protein most closely related to AlaDC. To investigate whether the new gene *AlaDC* originate from gene *SDC* and to determine the biochemical properties of the two proteins from *Camellia sinensis*, the sequences of *CsAlaDC* and *CsSDC* were analyzed and the two proteins were over-expressed, purified, and characterized.

**Results:**

The results showed that exon-intron structures of *AlaDC* and *SDC* were quite similar and the protein sequences, encoded by the two genes, shared a high similarity of 85.1%, revealing that new gene *AlaDC* originated from *SDC* by gene duplication. CsAlaDC and CsSDC catalyzed the decarboxylation of alanine and serine, respectively. CsAlaDC and CsSDC exhibited the optimal activities at 45 °C (pH 8.0) and 40 °C (pH 7.0), respectively. CsAlaDC was stable under 30 °C (pH 7.0) and CsSDC was stable under 40 °C (pH 6.0–8.0). The activities of the two enzymes were greatly enhanced by the presence of pyridoxal-5′-phosphate. The specific activity of CsSDC (30,488 IU/mg) was 8.8-fold higher than that of CsAlaDC (3467 IU/mg).

**Conclusions:**

Comparing to CsAlaDC, its ancestral enzyme CsSDC exhibited a higher specific activity and a better thermal and pH stability, indicating that CsSDC acquired the optimized function after a longer evolutionary period. The biochemical properties of CsAlaDC might offer reference for theanine industrial production.

**Supplementary Information:**

The online version contains supplementary material available at 10.1186/s12896-021-00674-x.

## Background

Tea is one of the most widely consumed beverages in the world. The popularity of tea could be attributed to its unique favor and health benefits [1, 2], which is provided by the special metabolites produced by tea plants (*Camelllia sinensis*) [3, 4]. Theanine (γ-glutamylethylamine), one of the specific metabolites in tea plants, is a key chemical component affecting green tea quality [4–6]. It is the most abundant non-protein amino acid in tea and accounts for more than 50% of the total free amino acids in tea plants [7]. Theanine plays important roles in nitrogen metabolism, especially in nitrogen storage [8] and ammonia detoxification [9]. Theanine also plays a crucial role in health promotion in humans, including improving memory [3], promoting concentration and relaxation, and reducing blood-pressure and mental stress [10]. Thus, theanine has been industrially produced and commercially developed as an additive used for food and beverages.

Theanine is synthesized from glutamate (Glu) and ethylamine catalyzed by theanine synthetase (TS) mainly in the roots of tea plants [11–14]. Ethylamine is derived mainly from the alanine decarboxylation catalyzed by alanine decarboxylase (AlaDC) [12, 14–16]. A great amount of theanine is accumulated in *C. sinensis,* but little or no theanine in other species [17, 18]. A previous study revealed that the differential accumulation of theanine between *C. sinensis* and other species could be attributed to the availability of ethylamine [18], indicating that CsAlaDC was a crucial enzyme in theanine biosynthesis pathway. Up to now, the new gene *AlaDC* has been found and reported only in *C. sinensis* [16] but not in any other species. Based on the consistency of the distribution of theanine and new gene *AlaDC* among species, it seemed that the differential accumulation of theanine might be attributed to the origination of *AlaDC*.

Serine decarboxylase (SDC), belonging to the pyridoxal-5′-phosphate-dependent decarboxylase (PLP-deC) super family, has been found to be the protein most closely related to CsAlaDC [16]. Previous studies suggested that AlaDC [16] and SDC [19] catalyzed the decarboxylation of alanine (Ala) and serine (Ser), respectively. Ala is similar to Ser in the chemical structure with the side chain of Ala being a methyl (−CH_3_) and that of Ser being a hydroxymethyl (−CH_2_OH). Based on the high similarity of the two enzymes in the protein sequence and catalytic activity, we speculated that the new gene *AlaDC* might originate from *SDC*.

It has been reported that the crude enzyme extracted from tea seedlings had the AlaDC activity [15], however, the biochemical properties of the pure enzyme CsAlaDC have not been characterized due to the lack of *AlaDC* gene sequence information. The *SDC* gene in tea plants has not been identified previously. To determine the biochemical properties of the new enzyme CsAlaDC and its putative ancestral enzyme CsSDC, the coding sequences of the two proteins were inserted into expression vector pET-32a (+), over-expressed in *Escherichia coli* BL21 (DE3), purified, and characterized.

## Results and discussion

### New gene *AlaDC* originated from *SDC* by gene duplication

**Fig. 1:**
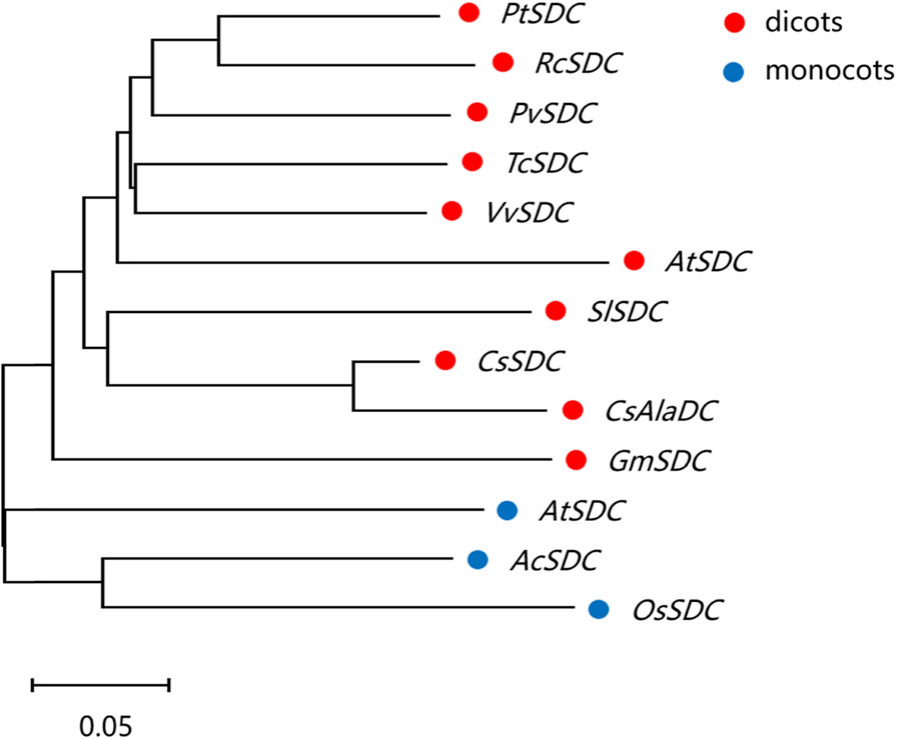
Minimum-Evolution phylogenetic analysis of *AlaDC* and *SDC* coding sequences from plants. The GenBank accession numbers of AlaDC and SDC genes used to build the phylogenetic tree are as follows: *PtSDC* (*Populus trichocarpa SDC,* XM_002306654.3); *RcSDC* (*Ricinus communis SDC,* XM_002532971.3); *PvSDC* (*Pistacia vera SDC,* XM_031428811.1); *TcSDC* (*Theobroma cacao SDC,* XM_007019401.2); VvSDC, (*Vitis vinifera SDC,* XM_002266362.4); *AtSDC* (*Arabidopsis thaliana SDC,* NM_03496.3); *SlSDC* (*Solanum lycopersicum SDC,* XM_004237726.4); *CsAlaDC* (*Camellia sinensis AlaDC,* MN241445.1); *GmSDC* (*Glycine max SDC,* XM_003547539.3); *AmtSDC* (*Amborella trichopoda SDC,* XM_006854458.3); *AcSDC* (*Ananas comosus SDC,* XM_020256259.1); *OsSDC* (*Oryza sativa SDC,* XM_015771197.1)

New genes were defined as those that are present in all members of a monophyletic group but absent from all outgroup species [20]. Our previous research has shown that AlaDC was a novel enzyme and SDC was the protein most closely related to AlaDC [16]. In this study, a BLAST search result showed that *AlaDC* gene was only found in tea plants, while the homologous genes of *SDC* were widely distributed in monocots and dicots (Fig. 1). Therefore, *AlaDC* might be a new gene originated *from SDC*.

**Fig. 2:**
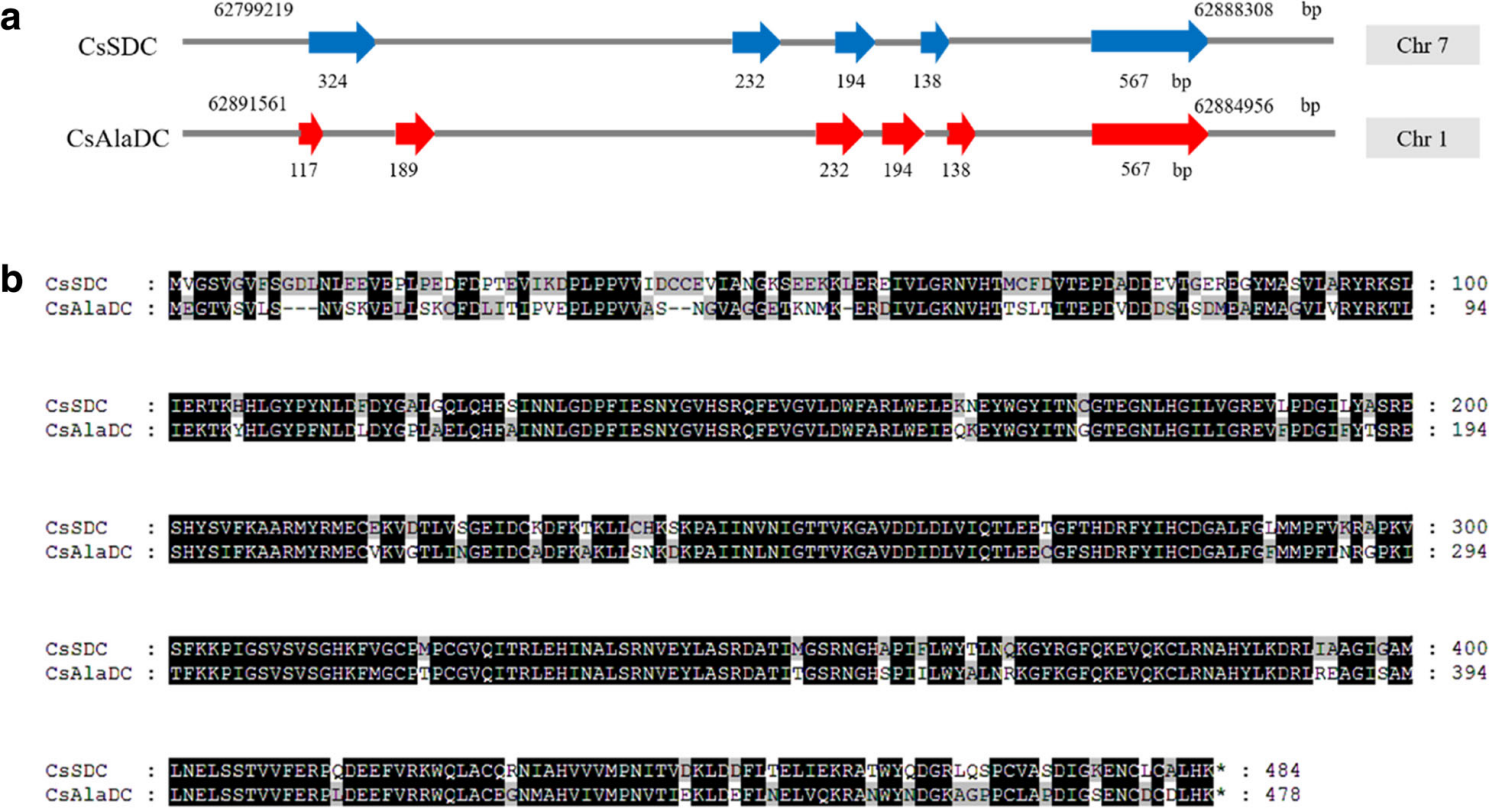
**a** Deduced exon-intron structures of *CsAlaDC* and *CsSDC*. **b** Pairwise alignment of the coding sequences of *CsAlaDC* and *CsSDC*

The *SDC* gene was amplified from the cDNA of *C. sinensis* cv. longjing 43. DNA sequencing results revealed that this gene was a possible *CsSDC* product of 1455 bp encoding a protein with 484 aa. Pairwise alignment of the two proteins showed that the amino acid sequence of CsSDC shared 74.0% identity and 85.1% similarity to that CsAlaDC (Fig. 2b). The exon-intron structures of *CsAlaDC* and *CsSDC* were deduced from comparisons of tea genomic sequence with the cDNA sequences of the two genes. Results suggested that the exon-intron structures of the two genes are also similar. The *CsAlaDC* gene, consisting of six exons, is located around 62884956–62891561 bp of chromosome 1, while the *CsSDC* gene, consisting of five exons, is located around 62799219–62888308 bp of chromosome 7 (Fig. 2a). The length of the last four exons of *CsSDC* are exactly same as that of *CsAlaDC*. However, the first exon of *CsSDC* seemed split into two exons (the first two exons of *CsAlaDC*) in *CsAlaDC* during evolution. The similarity of the two genes in exon-intron structure and coding sequence revealed that the new gene *AlaDC* originated from *SDC* by gene duplication.

### Overexpression and purification of CsAlaDC and CsSDC

**Fig. 3:**
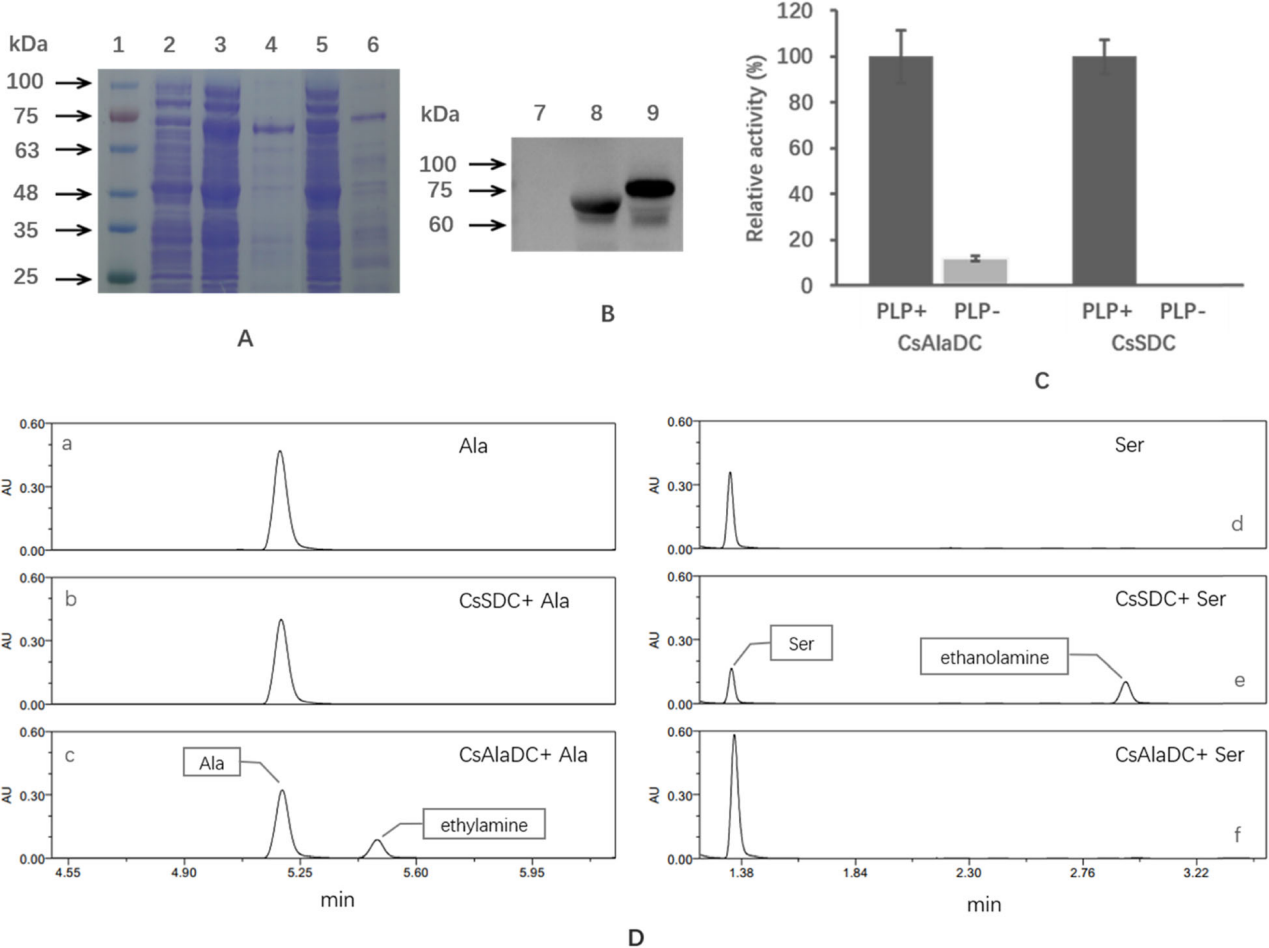
Purification and characterization of CsAlaDC and CsSDC. (**A**) SDS-PAGE analysis of CsAlaDC and CsSDC. Line 1, the molecular-weight marker; line 2, crude enzyme of the *E. coli BL21 (DE3)* transformed with pET-32a plasmids; line 3, crude enzyme of CsAlaDC; line 4, purified enzyme of CsAlaDC; line 5, crude enzyme of CsSDC; line 6, purified enzyme of CsSDC. (**B**) Western blot analysis of CsAlaDC and CsSDC. Line 7, crude enzyme of control; line 8, purified CsAlaDC with His-tag; line 9, purified CsSDC with His-tag. (**C**) Enzyme activity measurement of CsAlaDC and CsSDC in the reaction mixture with or without PLP. (**D**) Detection of enzyme activities of CsAlaDC and CsSDC by HPLC. (a) Reaction mixture containing Ala and the crude cell lysate of transformants with empty expression vector. (b and c) Reaction mixture containing Ala and the purified CsSDC (b) or CsAlaDC (c). (d–f) corresponding reaction mixture with Ala replaced by Ser

The genes *CsAlaDC* and *CsSDC* were inserted into the pET-32a (+) expression vector with a His_6_-tag at N-terminal. Two proteins were over-expressed in *E. coli* BL21 (DE3) cells and purified using Ni^+^ affinity chromatography. The purified proteins were identified by using 12% SDS-PAGE and western blot analysis (Fig. 3A and 3B). The molecular mass of CsSDC was estimated approximately as 75 kDa, which is slightly larger than that of CsAlaDC. The activities of the two enzymes catalyzing the decarboxylation of Ala (for CsAlaDC) and Ser (for CsSDC) were detected as indicators of enzyme purity (Fig. 3D). The specific activities of the crude proteins were 936 IU/mg (CsAlaDC) and 5980 IU/mg (CsSDC), respectively. After purification, the specific activities of the two enzymes increased to 3.1-fold (CsAlaDC) and 5.1-fold (CsSDC), respectively (shown in Table 1).

**Table 1:** Purification of recombinant protein CsAlaDC and CsSDC

Enzyme	Purification step	Total protein (mg)	Total activity (IU)	Specific activity (IU/mg)	Yield (%)	Purification fold
CsAlaDC	Crude protein	142.7	133,491	936	35.5	3.7
	Purified protein	13.7	47,383	3467		
CsSDC	Crude protein	130.6	780,720	5980	45.2	5.1
	Purified protein	11.6	352,756	30,488		

### Effect of PLP on enzyme activity

Previous studies reported that PLP was a cofactor of the PLP-deC, thus the effects of PLP on the enzymatic activities of CsAlaDC and CsSDC were examined. As shown in Fig. 3C, the activities of the two enzymes were significantly increased at the presence of PLP (0.1 mM). When there was no PLP in the reaction mixture, the activity of CsAlaDC was low and CsSDC was almost inactive. When PLP (0.1 mM) was added to the reaction mixture, the activity of CsAlaDC increased 7.37 folds and CsSDC was rapidly activated. This result indicated that the activities of CsAlaDC and CsSDC are significantly dependent on the presence of PLP.

The increase in enzyme activities at the presence of PLP was also observed in other PLP-dependent amino acid decarboxylases (AADs). For example, in PLP-deficient mixture, the activity of SDC in *Arabidopsis thaliana* was low, whereas after the addition of PLP, the enzyme activity increased more than 30 folds [19]. Human histidine decarboxylase (HDC) in PLP-deficient mixture showed only 30% of normal activity (in PLP-present mixture) and the addition of PLP restored the activity to normal level [21]. The activities of arginine decarboxylase (ADC) [22] and tyrosine decarboxylase (TYDC) were also significantly increased when PLP was added to the reaction mixture [23].

A previous study showed that the AlaDC activity of crude protein extracted from tea seedlings was not activated by PLP [15]. That was inconsistent with the results obtained in the present study and with the properties of other AADs reported previously. Previous research revealed that, at the presence of PLP, the PLP-dependent decarboxylase shifted from an unstable open conformation to a stable closed conformation by binding to PLP [24, 25]. The AlaDC activity measured in the previous study was the activity of crude protein extracted from tea seedlings, and the protein might had already bound to PLP required for the decarboxylation reaction before it was extracted. Thus, even the addition of PLP to the reaction system had little effect on enzyme activity.

### Substrate specificity

To investigate the substrate specificity of CsAlaDC and CsSDC, the activities of the two enzymes were detected with various substrates, including Ala, Ser, histidine, glutamate, arginine, tyrosine, and tryptophan. The corresponding amines produced in the reaction mixture were detected by UPLC. The results showed that CsAlaDC could only catalyze the decarboxylation of Ala, but not Ser or other amino acids. CsSDC could only catalyze the decarboxylation reaction of Ser, but not Ala or other amino acids (as shown in Fig. 3D). Therefore, CsAlaDC and CsSDC had completely different biochemical functions and were responsible for catalyzing the decarboxylation of Ala and Ser, respectively.

### Optimal pH for enzyme activity and stability

**Fig. 4:**
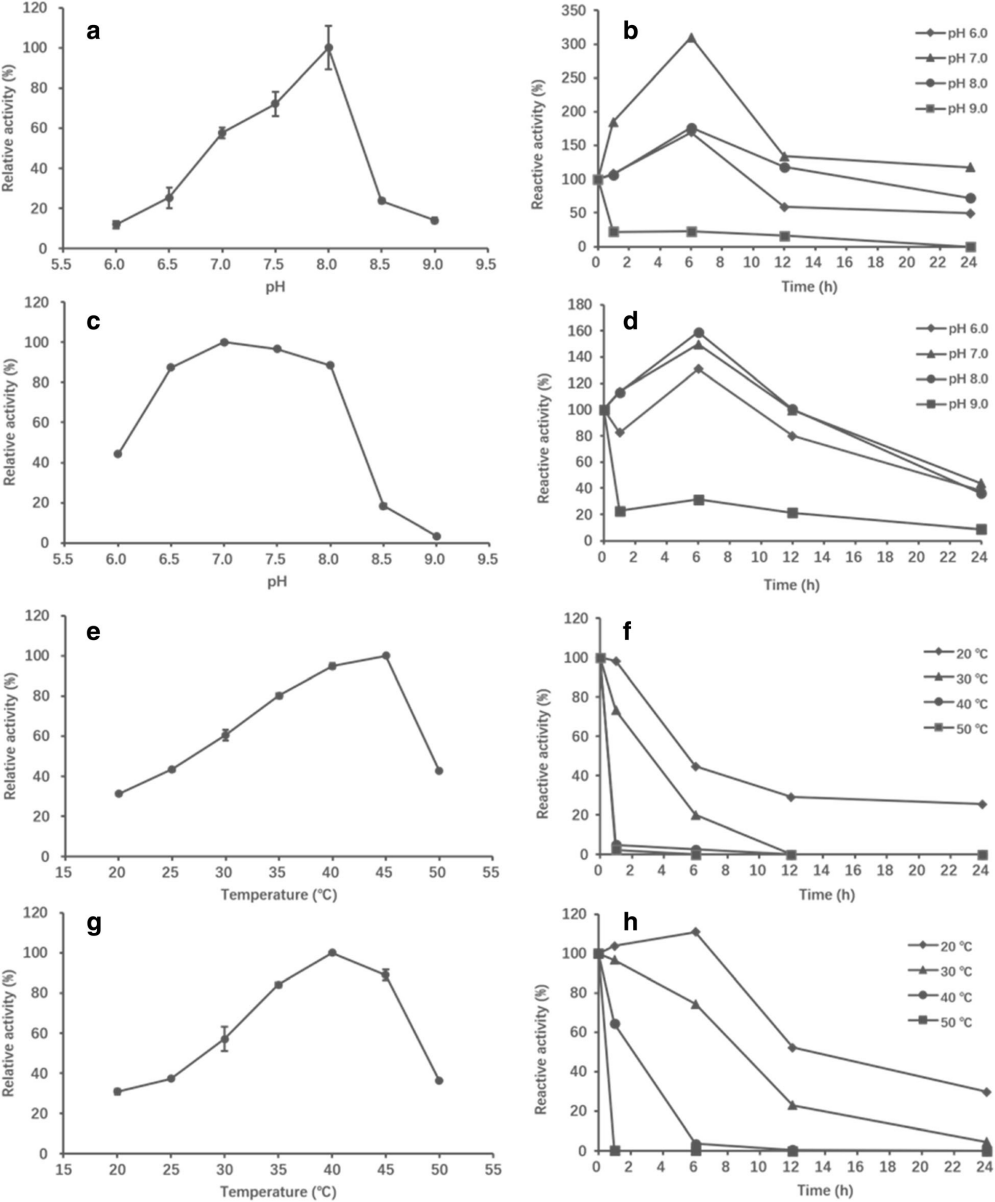
Effect of pH and temperature on enzyme activity and stability. **a** Effect of pH on CsAlaDC activity; **b** The pH stability of CsAlaDC after 1–24 h incubation at 4 °C and pH of 6.0–9.0; **c** Effect of pH on CsSDC activity; **d** The pH stability of CsAlaDC. **e** Effect of temperature on CsAlaDC activity; **f** Thermal stability of CsAlaDC after 1–24 h incubation within the range of 20–50 °C; **g** Effect of temperature on CsSDC activity; **h** Thermal stability of CsAlaDC

The optimal pH of CsAlaDC and CsSDC was found to be 8.0 and 7.0, respectively (Fig. 4a and 4c). CsAlaDC maintained high enzyme activity only in a very narrow pH range (Fig. 4a). When the pH value dropped from 8.0 to 7.0, the enzyme activity of CsAlaDC decreased to 57.8% of its maximum activity, while at pH 6.5 and 8.5, its activity decreased to 25.4 and 23.7%, respectively. Whereas, CsSDC maintained high enzyme activity (> 85%) over a wide pH range (pH 6.5–8.0), indicating that CsSDC was more adaptable to environment changes than CsAlaDC. A previous study suggested that the maximum AlaDC activity of the crude protein extracted from tea seedlings was detected at pH 6.25 [15], which was different from the results obtained in the present study. This might be attributed to the fact that the crude protein extracted from tea seedlings might contain other enzymes or components, resulting in differences in the measured optimum pH of the crude enzyme and pure enzyme CsAlaDC.

The optimal pH for other AADs was between 3.8 and 8.5. Some enzymes had an optimum pH similar to that of CsAlaDC and CsSDC, such as AtSDC [19], ADC (pH 6.5–8.5) [22, 26, 27] and tyrosine decarboxylase (TDC) (pH 7.0–8.5) [23, 28]. Some decarboxylases showed the best enzymatic activity under weakly acidic conditions, such as HDC (pH 4.4–7.0) [29], while others have a wide pH range, such as glutamate decarboxylase (GAD) (pH 3.8–8.0) [30–33].

To investigate the stability of CsAlaDC and CsSDC under different pH conditions, the two enzymes were treated in pH 6.0–9.0 buffer (4 °C) for 1, 6, 12 and 24 h, respectively, and the residual enzyme activities were measured. The results showed that the two enzymes were stable in pH 6.0–8.0, and the enzyme activities decreased rapidly when the pH value reached 9.0 (Fig. 4b and 4d). Surprisingly, the enzymatic activity of CsAlaDC and CsSDC significantly increased within 6 h under suitable pH conditions (pH 6.0–8.0). After 6-h incubation at pH 7.0, the enzyme activities of CsAlaDC and CsSDC increased to 3.1 folds and 1.5 folds, respectively. This might be due to the pre-addition of coenzyme PLP to buffers of different pHs, where PLP bind to the decarboxylases during incubations, resulting in higher enzyme activities after treatments. Although CsAlaDC showed the best enzyme activity at pH 8.0, the enzyme was more stable at pH 7.0. Therefore, the reaction conditions at pH 7.0 would be favorable for the industrial production of theanine. The AADs with similar pH stability to CsAlaDC and CsSDC were ADC (pH 7.0–7.5) [22] and TDC [34] (pH 7.4). Whereas HDC (pH 4.5) [29] and GAD (pH 5.0–7.0) [32, 33] were more stable in acidic environments. This study revealed that CsAlaDC was stable at pH 7.0 and CsSDC was stable at pH 6.0–8.0, and the pH stability of the two enzymes might offer reference for theanine industrial production and reflect their adaptation to intracellular pH of *C. sinensis*.

### Optimal temperature for enzyme activity and stability

The activities of CsAlaDC and CsSDC increased with increasing temperature below 40 °C. The two enzymes reached their maximum activities at 45 °C (for CsAlaDC) and 40 °C (for CsSDC), respectively (Fig. 4e and 4g). While at 50 °C, the activities of the two enzymes decreased rapidly to 42.6% (for CsAlaDC) and 36.2% (for CsSDC) of their maximum activities, respectively.

The optimum temperature for the other AADs was between 30 °C and 70 °C. Each decarboxylase had a different optimum temperature depending on the species of origin. For example, GADs in *Lactobacillus brevis* and *Escherichia coli* had lower optimum temperature of 30 °C and 37 °C, respectively. The optimal temperature of GADs in *Enterrococcus raffinosus*, *Lactobacillus paracasei* and *Streptococcus salivarius*, were between 45 °C and 55 °C, while in *Aspergillus Oryzae* was up to 60 °C [30]. Even for GADs in different strains of *Lacto bacillus*, the optimal reaction temperatures varied (30–55 °C) [31]. Similarly, the optimal reaction temperatures of ADCs from different strains were varied. The optimal temperatures of ADCs in *Bacillus subtilis* and *Yersinia pestis* was 37 °C, in *E. coli* was 50 °C, in *Clostridium difficile* was 60 °C and in *Chloroflexus aurantiacus* and *Slufolobus solfataricus* was up to 70 °C [22].

The thermal stability of CsAlaDC and CsSDC at 20 °C to 50 °C was investigated. The results showed that CsSDC was more thermally stable than CsAlaDC (Fig. 4f and 4h). There was no detectable activity of both enzymes after 1-h incubation at 50 °C. After 1-h incubation at 40 °C, CsAlaDC lost almost all of its activity, while CsSDC still maintained 64.4% of its activity. After 1-h incubation at 30 °C, the residual enzyme activity of CsAlaDC was 73.3%, and that of CsSDC was 96.8%. After 1-h incubation at 20 °C, both enzymes maintained almost full enzyme activity. After 12-h incubation at 30 °C, CsAlaDC lost all its enzyme activity, and CsSDC maintained 23.0%. After 24-h incubation at 20 °C, the residual activity of both enzymes was 25.4% (CsAlaDC) and 29.8% (CsSDC), respectively.

Other AADs seemed to be more thermally stable than CsSDC and CsAlaDC. For instance, after 1-h incubation at 40 °C, ADC from *E. coli* could maintain 78.6% of its initial activity, and TYDC from *Lactobacillus subtilis* could maintain more than 90% [34]. ErGAD maintained more than 85% of its initial activity after 5-h incubation at 50 °C [30], and GAD from *Bacillus megaterium* maintained more than 75% activity after 6-h incubation at 40 °C [32].

The maximal activity of CsAlaDC and CsSDC was detected at 45 °C and 40 °C, respectively (Fig. 4e and 4g). However, the two enzymes showed poor stability at temperature above 40 °C. Since temperature up to 40 °C led to the decrease in enzyme stability, 30–35 °C was recommended as a suitable condition in industrial application.

### Kinetic parameters

**Fig. 5:**
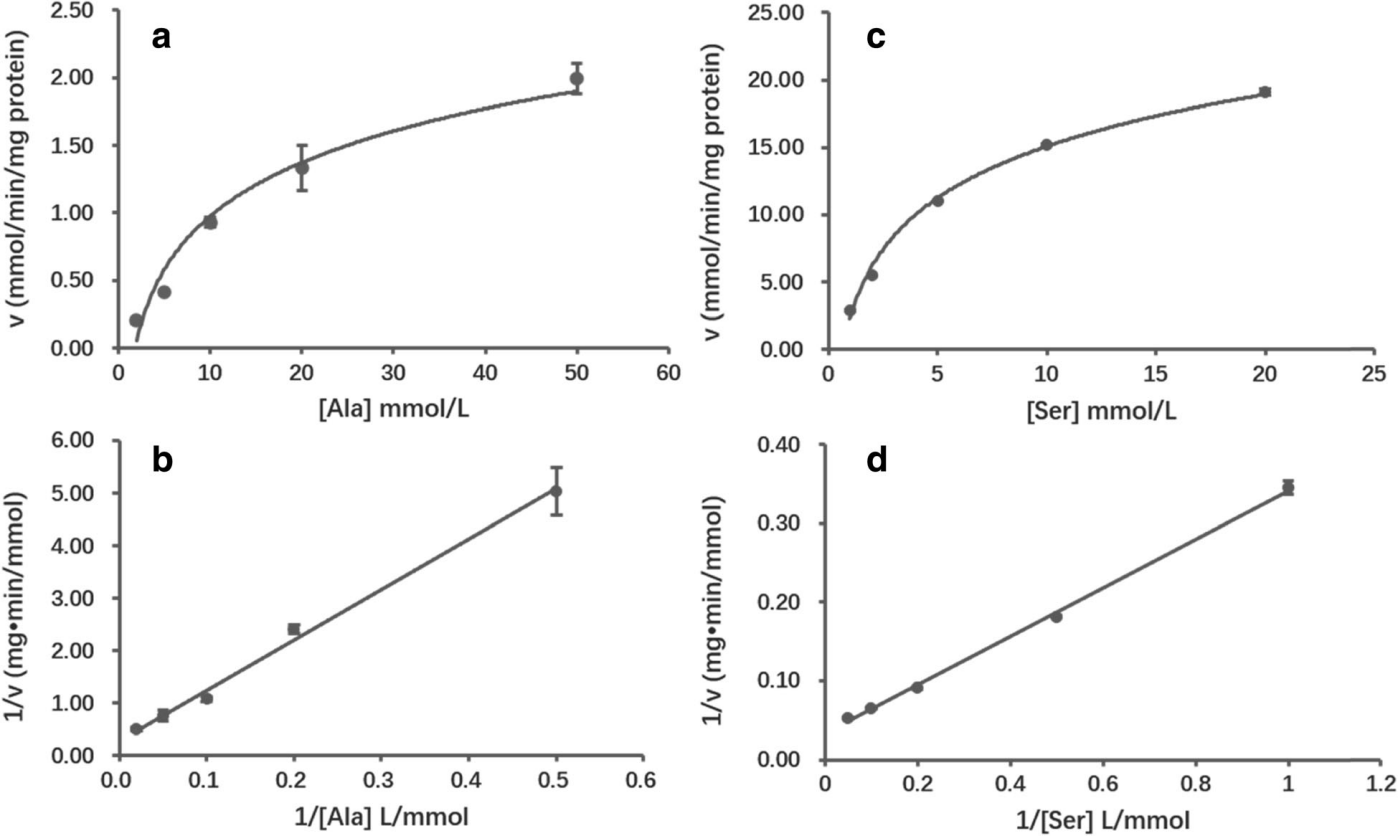
Catalytic velocity of CsAlaDC and CsSDC at various substrate concentrations. **a** velocity of CsAlaDC dependent on alanine concentrations. **b** Lineweaver-Burk plot of CsAlaDC; **c** velocity of CsSDC dependent on serine concentrations; **d** Lineweaver-Burk plot of CsSDC

The kinetic parameters of CsAlaDC and CsSDC were determined by Lineweaver-Burk Plots (Fig. 5b and 5d) using corresponding substrate (Fig. 5a and c). The V_max_ of CsSDC (30.49 mmol•min^− 1^ mg^− 1^) was 8.8-fold higher than that of CsAlaDC (3.47 mmol•min^− 1^ mg^− 1^) (shown in Table 2), indicating that the new enzyme CsAlaDC had a much lower catalytic efficiency than its ancestral enzyme CsSDC. The K_m_ value of CsAlaDC (33.25 mM) was much higher than that of CsSDC (9.42 mM), which was close to the K_m_ value of AtSDC (10 mM) reported in the previous study [19]. This result suggested that the substrate affinity of CsAlaDC was lower than that of CsSDC and AtSDC. Among other AADs, except GADs (0.045–22.9 mM) [30–33], most AADs were reported to have a K_m_ value lower than that of CsAlaDC (33.25 mM) and CsSDC (9.42 mM), including HDC (0.2–0.8 mM) [29, 35], ADC (0.03–5.6 mM) [22, 26, 27] and TDC (0.25–0.6 mM) [23, 28, 34].

**Table 2:** Kinetic parameters of CsAlaDC and CsSDC

Enzyme	Substrate	K_m_ (mM)	V_max_ (mmol•min^−1^ mg^− 1^)
CsAlaDC	alanine	33.25	3.47
CsSDC	serine	9.42	30.49

A recent study also measured the activity of CsAlaDC expressed by prokaryotic system, and the results suggested that the protein had no AlaDC activity [36]. There are four possible reasons for these results. Firstly, the amino acid sequence of CsAlaDC used in that study was not identical to the sequence used in the present and in our previous study. The coding sequence of *CsAlaDC* gene was submitted to GenBank (accession number: MN241445.1). Secondly, as reported in the present study, the enzymatic activity of CsAlaDC was significantly dependent on the presence of PLP, which was absent from the reaction system in that study. Thirdly, the AlaDC activity was assayed by measuring the substrate (Ala) consumption in that study. That method might not be accurate enough due to the relatively low activity of new enzyme CsAlaDC. Finally, compared with the mature enzyme CsSDC, the new enzyme CsAlaDC had lower enzymatic activity, was unstable and easily deactivated, therefore, before using CsAlaDC for theanine production, the expression and reaction conditions of the enzyme need to be optimized, as reported in this study.

## Conclusion

In conclusion, an SDC from *C. sinensis* was identified as the ancestral enzyme of AlaDC. The high similarity of *CsAlaDC* and *CsSDC* in gene structures and their encoded protein sequences revealed that the new gene *AlaDC* originated from *SDC* by gene duplication. CsSDC and CsAlaDC are enzymes that specifically catalyze the decarboxylation of Ser and Ala, respectively. The activities of the two enzymes were greatly enhanced by the presence of pyridoxal-5′-phosphate. Comparing to CsAlaDC, its ancestral enzyme CsSDC exhibited a higher specific activity and a better thermal and pH stability. The specific activity of CsSDC (30,488 IU/mg) was 8.8-fold higher than that of CsAlaDC (3467 IU/mg). CsAlaDC was stable under 30 °C (pH 7.0) and CsSDC was stable under 40 °C (p H 6.0–8.0). This study characterized the biochemical properties of CsAlaDC and its ancestral enzyme CsSDC, further efforts should be made to find the active sites or the key residues affecting the substrate specificity of the two enzymes.

## Methods

### Sequence analysis of CsAlaDC and CsSDC

The *CsAlaDC* gene used was from the recombinant plasmid previously conserved in our laboratory. The gene sequence of *CsSDC* was found from the transcriptome of *C. sinensis* and the gene was isolated from the cDNA of *C. sinensis* cv. longjing 43. The PCR products were confirmed by 1.2% agarose gel electrophoresis and DNA sequencing. Homologous genes from different plant species were obtained from non-redundant sequence database of transcripts. The phylogenetic tree of these AADs was constructed using the Minimum-Evolution method with MEGA X. To determine the exon-intron structures of *CsAlaDC* and *CsSDC*, the cDNA sequences of those two genes were aligned to the genomic sequence of *C. sinensis* [37]. Alignment of the protein sequences of CsAlaDC and CsSDC was conducted by MEGA X.

### Overexpression and purification of recombinant protein

The genes *CsAlaDC* and *CsSDC* were inserted into expression vector pET-32a (+) with a His_6_-tag at N-terminal and the recombinant plasmids were transferred into *E. coli* BL21 (DE3) (Shanghai Weidi biotechnology, China), grown in LB medium containing 100 μg/mL ampicillin at 37 °C. When the optical density (OD) at 600 nm reached 0.4–0.6, IPTG (iso-propyl β-D-1-thiogalactopyranoside) (Solarbio, China) was added with a final concentration of 0.1 mM, and the cells were cultured at 16 °C for 16 h, harvested by centrifugation at 4 °C and 4000 rpm for 30 min, washed with distilled water, and resuspended in phosphate-buffered saline (PBS, pH 7.4) (Solarbio, China). The recombinant proteins were extracted through cell ultrasonication disruption (200 W, 5 s, 5 s, 20 min) and centrifugation (at 4 °C and 12,000 rpm, 30 min). The supernatants were purified with a Ni-Agarose resin column (CWBIO, China). The purified proteins were detected by 12.5% sodium dodecyl sulfate-polyacrylamide gel electrophoresis (SDS-PAGE) (Bio-Rad, USA). The presence of His-tagged CsAlaDC and CsSDC were verified by western blot analysis using an His-tag monoclonal antibody (Proteintech, USA) and an anti-mouse lgG as the primary and secondary antibodies. Protein concentration was measured by Bradford method with bovine serum albumin (BSA) as a standard.

### Enzyme activity assays

Decarboxylase activity was measured with detecting products (ethylamine or ethanolamine) in Waters Acquit ultraperformance liquid chromatography (UPLC) system. The 100 μL reaction mixture, containing 20 mM substrate (Ala or Ser), 100 mM potassium phosphate 0.1 mM pyridoxal-5′-phosphate (PLP), 5 mM L-dithiothreitol, 1 mM K_2_EDTA (Sangon Biotech, China), 10% glycerol, and 20 μL purified enzyme (~ 12 μg protein), was prepared and incubated at standard conditions (45 °C and pH 8.0 for CsAlaDC, 40 °C and pH 7.0 for CsSDC) for 15 min. Then, the reaction was stopped with 20 μL of 10% trichloroacetic acid. After derivatization with AccQ·Tag reagents (Waters, USA), the reaction products in the mixture were analyzed with UPLC. All enzymatic assays were performed in triplicate.

### Effect of PLP on enzyme activity

To investigate the effect of PLP on enzyme activity, the enzyme activity of the reaction mixture was measured at standard conditions with 20 mM substrate at the presence or absence of 0.1 mM PLP. The enzyme activity of reaction mixture containing PLP was taken as 100%.

### Substrate specificity of CsAlaDC and CsSDC

The substrate specificity was measured at standard conditions with 5 mM substrates including serine, alanine, histidine, arginine, glutamate, tyrosine and tryptophan, respectively.

### Optimal pH and pH stability

The optimal pHs of purified enzymes were determined by using reaction mixture with pH ranging from 6.0 to 9.0. To examine pH stability of the enzymes, the residual activities were measured at standard conditions after pre-incubation at pH value range of 6.0–9.0 for 1, 6, 12 and 24 h, respectively. The enzyme activity at optimal pH was taken as 100%, and the percentage of the residual activities at different pH values against enzyme activity at optimal pH were calculated as relative activities.

### Optimal temperature and thermal stability

The optimal temperatures of purified enzymes were determined by performing the reactions at temperature range from 20 °C to 50 °C. To investigate thermal stability of the two enzymes, residual activities were measured at standard conditions after pre-incubation within temperature range of 20–50 °C for 1, 6, 12 and 24 h, respectively. The enzyme activity at optimal temperature was taken as 100%, and the percentage of the residual activities at different temperature values against the enzyme activity at optimal temperature were calculated as relative activities.

### Kinetic parameters

Kinetic parameters of the two enzymes were measured at standard conditions by applying different concentrations of Ala and Ser in the reaction mixtures. The concentrations of Ala and Ser ranged from 1 mM to100 mM. According to Lineweaver-Burk plots, K_m_ and V_max_ values were calculated.

## Supplementary Information

**Additional file 1.**

## Data Availability

All data generated or analyzed during this study are included in this published article [and its supplementary information files].

## Abbreviations

AAD: Amino acid decarboxylase
ADC: Arginine decarboxylase
Ala: Alanine
AlaDC: Alanine decarboxylase
BSA: Bovine serum albumin
GAD: Glutamate decarboxylase
Glu: Glutamate
HDC: Histidine decarboxylase
IPTG: Iso-propyl β-D-1-thiogalactopyranoside
OD: Optical density
PBS: Phosphate-buffered saline
PLP: Pyridoxal-5′-phosphate
PLP-deC: Pyridoxal-5′-phosphate-dependent decarboxylase
SDC: Serine decarboxylase
SDS-PAGE: Sodium dodecyl sulfate-polyacrylamide gel electrophoresis
Ser: Serine
TDC: Tyrosine decarboxylase
TS: Theanine synthetase
TYDC: Tyrosine decarboxylase
UPLC: Ultraperformance liquid chromatography
