# Supplementary material for: Biochemical characterization of specific Alanine Decarboxylase (AlaDC) and its ancestral enzyme Serine Decarboxylase (SDC) in tea plants (Camellia sinensis)
Source: BMC Biotechnol. 2021 Mar 1;21:17. doi: 10.1186/s12896-021-00674-x (PMC7923638; doi:10.1186/s12896-021-00674-x)
Supplement: Supplementary file 1 — Additional file 1. [file 12896_2021_674_MOESM1_ESM.pdf]

>CsSDC
ATGGTGGGAAGTGTTGGGGTGTTTTCTGGCGATCTCAATCTCGAAGAGGTTGAGCCTT
TGCCGGAGGATTTTGATCCAACTGAAGTGATTAAAGATCCTCTGCCACCGGTGGTGATT
GATTGCTGCGAAGTCATCGCCAATGGCAAAAGTGAAGAAAAGAAGCTGGAAAGGGAG
ATTGTGCTTGGGAGGAATGTGCACACAATGTGCTTTGATGTCACCGAGCCCGATGCCG
ACGATGAGGTCACCGGAGAGAGGGAAGGCTATATGGCTAGTGTGTTGGCTAGATATAG
AAAGTCTTTGATTGAGAGGACTAAGCACCATTTGGGGTACCCCTACAATCTTGATTTCG
ATTATGGTGCTCTGGGACAGTTACAACATTTCTCCATTAACAATCTTGGTGATCCATTTA
TAGAGAGCAACTATGGAGTCCATTCCAGGCAGTTTGAAGTGGGCGTATTGGATTGGTTT
GCCCGCCTATGGGAACTTGAGAAGAATGAATATTGGGGCTACATTACAAATTGTGGAAC
AGAAGGCAATCTTCATGGTATATTAGTCGGGAGGGAAGTGCTTCCAGATGGCATTCTGT
ATGCCTCACGTGAATCACATTATTCTGTTTTCAAAGCTGCACGAATGTACAGGATGGAA
TGTGAAAAGGTCGACACTTTGGTCTCAGGTGAAATTGATTGCAAGGATTTCAAAACTA
AACTCCTTTGCCACAAGAGCAAGCCAGCAATTATCAACGTAAACATTGGCACAACTGT
TAAGGGAGCTGTTGATGATCTAGATCTGGTTATACAGACCCTCGAAGAAACAGGATTCA
CACATGACCGGTTCTACATTCACTGCGATGGGGCTCTATTTGGTCTCATGATGCCTTTTG
TAAAACGTGCACCGAAAGTTTCTTTCAAGAAGCCTATAGGAAGCGTGAGTGTTTCTGG
CCACAAGTTTGTGGGATGCCCTATGCCTTGTGGCGTTCAAATAACAAGATTGGAGCAC
ATTAATGCCCTATCAAGGAACGTGGAGTATCTTGCTTCTAGAGATGCAACTATTATGGGA
AGCAGAAATGGCCATGCACCAATCTTCCTCTGGTACACCTTAAACCAGAAAGGGTACA
GAGGGTTCCAGAAAGAAGTACAAAAATGCCTCAGGAATGCACACTACTTGAAGGACC
GCCTCATAGCAGCTGGCATTGGAGCAATGCTCAACGAACTCAGCAGCACAGTTGTGTT
TGAGCGCCCACAAGATGAGGAGTTTGTTCGCAAGTGGCAGCTCGCTTGCCAAAGGAA
CATTGCACATGTGGTGGTCATGCCCAACATTACCGTCGATAAGCTTGATGATTTCTTGAC
CGAACTGATTGAGAAACGTGCCACTTGGTATCAGGATGGGAGGCTTCAATCTCCTTGT
GTTGCTTCAGATATAGGTAAGGAAAACTGTCTTTGTGCACTACACAAGTGA
